# Laryngeal and vocal alterations after thyroidectomy^☆^

**DOI:** 10.1016/j.bjorl.2017.08.015

**Published:** 2017-09-21

**Authors:** Renata Mizusaki Iyomasa, José Vicente Tagliarini, Sérgio Augusto Rodrigues, Elaine Lara Mendes Tavares, Regina Helena Garcia Martins

**Affiliations:** a Universidade Estadual Paulista “Júlio de Mesquita Filho” (UNESP), Disciplina de Otorrinolaringologia e Cirurgia de Cabeça e Pescoço, Botucatu, SP, Brazil; b Universidade Estadual Paulista “Júlio de Mesquita Filho” (UNESP), Instituto de Biociências, Botucatu, SP, Brazil

**Keywords:** Thyroidectomy, Dysphonia, Laryngeal paralysis, Hoarseness, Acoustic analysis, Tireoidectomia, Disfonia, Paralisia laríngea, Rouquidão, Análise acústica

## Abstract

**Introduction:**

Dysphonia is a common symptom after thyroidectomy.

**Objective:**

To analyze the vocal symptoms, auditory-perceptual and acoustic vocal, videolaryngoscopy, the surgical procedures and histopathological findings in patients undergoing thyroidectomy.

**Methods:**

Prospective study. Patients submitted to thyroidectomy were evaluated as follows: anamnesis, laryngoscopy, and acoustic vocal assessments. Moments: pre-operative, 1st post (15 days), 2nd post (1 month), 3rd post (3 months), and 4th post (6 months).

**Results:**

Among the 151 patients (130 women; 21 men). Type of surgery: lobectomy + isthmectomy *n* = 40, total thyroidectomy *n* = 88, thyroidectomy + lymph node dissection *n* = 23. Vocal symptoms were reported by 42 patients in the 1st post (27.8%) decreasing to 7.2% after 6 months. In the acoustic analysis, f0 and APQ were decreased in women. Videolaryngoscopies showed that 144 patients (95.3%) had normal exams in the preoperative moment. Vocal fold palsies were diagnosed in 34 paralyzes at the 1st post, 32 recurrent laryngeal nerve (lobectomy + isthmectomy *n* = 6; total thyroidectomy *n* = 17; thyroidectomy + lymph node dissection *n* = 9) and 2 superior laryngeal nerve (lobectomy + isthmectomy *n* = 1; Total thyroidectomy + lymph node dissection *n* = 1). After 6 months, 10 patients persisted with paralysis of the recurrent laryngeal nerve (6.6%). Histopathology and correlation with vocal fold palsy: colloid nodular goiter (*n* = 76; palsy *n* = 13), thyroiditis (*n* = 8; palsy *n* = 0), and carcinoma (*n* = 67; palsy *n* = 21).

**Conclusion:**

Vocal symptoms, reported by 27.8% of the patients on the 1st post decreased to 7% in 6 months. In the acoustic analysis, f0 and APQ were decreased. Transient paralysis of the vocal folds secondary to recurrent and superior laryngeal nerve injury occurred in, respectively, 21% and 1.3% of the patients, decreasing to 6.6% and 0% after 6 months.

## Resumo

**Introdução:**

A disfonia é um sintoma comum após a tireoidectomia.

**Objetivo:**

Analisar os sintomas vocais, auditivo-perceptivos e acústica vocal, videolaringoscopia, procedimento cirúrgico e achados histopatológicos em pacientes submetidos à tireoidectomia.

**Método:**

Estudo prospectivo. Pacientes submetidos à tireoidectomia foram avaliados da seguinte forma: anamnese, laringoscopia e avaliações vocais acústicas. Momentos: pré-operatório, 1ª avaliação pós (15 dias), 2ª avaliação pós (1 mês), 3ª avaliação pós (3 meses) e 4ª avaliação pós-operatória (6 meses).

**Resultados:**

Dos 151 pacientes, 130 eram mulheres e 21, homens. Tipos de cirurgia: lobectomia + istmectomia n = 40, tireoidectomia total n = 88, tireoidectomia + dissecção de linfonodo n = 23. Sintomas vocais foram relatados por 42 pacientes na 1ª avaliação pós-operatória (27,8%), reduzidos para 7,2% após 6 meses. Na análise acústica, f0 e APQ estavam diminuídos nas mulheres. As videolaringoscopias mostraram que 144 pacientes (95,3%) tiveram exames normais no momento pré-operatório. Paralisia das cordas vocais foi diagnosticada em 34 pacientes na 1ª avaliação pós-operatória, 32 do nervo laríngeo recorrente (lobectomia + istmectomia - n = 6; tireoidectomia total - n = 17; tireoidectomia total+dissecção de linfonodos - n = 9) e 2 do nervo laríngeo superior (lobectomia + istmectomia - n = 1; tireoidectomia total + dissecção de linfonodos - n = 1). Após 6 meses, 10 pacientes persistiram com paralisia do nervo laríngeo recorrente (6,6%). Histopatologia e correlação com paralisia das cordas vocais: bócio coloide nodular (n = 76; paralisia n = 13), tireoidite (n = 8; paralisia n = 0) e carcinoma (n = 67; paralisia n = 21).

**Conclusão:**

Os sintomas vocais, relatados por 27,8% dos pacientes na 1ª avaliação pós-operatória, diminuíram para 7% em 6 meses. Na análise acústica, f0 e APQ diminuíram. A paralisia transitória de cordas vocais secundária à lesão do nervo laríngeo recorrente e nervo laríngeo superior ocorreu, respectivamente, em 21% e 1,3% dos pacientes, reduziu-se para 6,6% e 0% após 6 meses.

## Introduction

Total (TT) or partial thyroidectomy (PT) may result in vocal impairment due to extrinsic compression of the gland on the larynx, endotracheal intubation, dissection of the cervical muscles, hematomas, and damage to the laryngeal nerves. Dysphonia may occur in up to 90% of patients, especially in the immediate postoperative period and persist for three to 6 months in 11–15% of cases.^1^ The recurrent laryngeal nerve (RLN) as well as the external branch of the superior laryngeal nerve (SLN) can be temporarily or permanently injured. The estimated rates of injury to the SLN vary from 0.3% to 13% and to the RLN from 5% to 10%, being temporary in 5% and permanent in 0.5–2%.^2^

Damage to the RLN is the main cause of dysphonia after thyroidectomy.^3^, ^4^, ^5^ The recurrent laryngeal nerve has several extralaryngeal branches and direct relation with the inferior thyroid artery, at superficial and deep level, and may be injured as the artery is divided. Paralysis of the RLN results in immobility of the vocal fold on the affected side, causing hoarseness and vocal fatigue as a consequence of glottic insufficiency.^6^, ^7^ The superior laryngeal nerve may be affected during thyroidectomy when the superior thyroid artery is divided or after local cauterization,^6^ resulting in a less tense vocal fold by decreased activity of the cricothyroid muscle (CT). Under these conditions, the vocal ability of a rapid change in register is impaired, as well as the emission of high pitch sounds and pitch maintenance. However some authors point out changes in vocal patterns in 14–30% of patients after thyroidectomy, even without nerve damage.^8^ The causes include laryngotracheal fixation by adherence to pre-tracheal muscles, injury to the perithyroid neural plexus, trauma from intubation, surgical trauma of the CT or cricothyroid junction, and changes in the vascular supply and venous and lymphatic drainage of the larynx. Most of these laryngeal alterations are self-limited.^9^, ^10^

In a study that included 54 patients who underwent TT, Stojadinovic et al.^10^ analyzed the voices of the patients before and after surgery and identified only one case of SLN injury and no cases of RLN paralysis. However, 30% of patients presented dysphonia in the 1st postoperative day and 14% were still symptomatic after 3 months. In addition, 84% of patients had alterations in at least one acoustic parameter in the immediate postoperative period.

Patients with thyroid cancer have higher risk of laryngeal nerve impairment by tumor infiltration, and may present preoperative vocal symptoms. Roh et al.^11^ in a study that included 319 patients with papillary thyroid carcinoma (256 had total thyroidectomy, 42 lobectomy, and 21 were re-operated for recurrent carcinoma) identified 14 patients with preoperative vocal fold paralysis and 15 with postoperative paralysis. In the latter, the paralysis was temporary in 4.6% and permanent in 1.3%. The authors emphasized that vocal fold paralysis may go unnoticed by the patient in up to 50% of cases, especially when it starts in a slow and insidious manner, reinforcing the importance of videolaryngoscopy before and after surgery.

The objective of this study was to analyze the presence of vocal symptoms, acoustic vocal characteristics, and videolaryngoscopy findings in patients undergoing thyroidectomy, relating them to the type of surgery and histopathology.

## Methods

All patients seen at a University clinic for thyroid disease submitted to thyroidectomy from 2012 to 2015 were invited to participate in the study. The study was approved by the Institutional Committee for Ethics in Human Research and the patients signed a free, prior, and informed consent.

Exclusion criteria: patients with previous thyroid, neck surgery or benign laryngeal lesions, previous vocal folds paralysis, neuromuscular disorders that compromise laryngeal structures, subjects with lung diseases or history of prolonged intubation.

Demographic data, voice symptoms, respiratory disorders, previous thyroid surgeries, type of surgical procedure and histopathology were recorded. The patients were submitted to laryngoscopy using a rigid telescope (70°, 8 mm, brand Asap, Germany) or nasal endoscopes (diameter 3.5 mm, Olympus, Japan) coupled with image capturing conjugated system (multifunctional video system type XE-50, Eco V 50W X – TFT/USB – ILO ELECTRONIC GnbH, Carl – Zeiss, Germany).

The MDVP system (Multi Dimensional Voice Program – Multi Speech 3700, model 5105, Kay Elemetrics Corporation, USA) and a headset microphone (Shure, São Paulo, SP, Brazil) connected to the soundboard (Behringer Xenyx 502 model, Germany) were used for acoustic vocal analysis during sustained emission of the /a/ vowel, maintaining comfortable pitch. The initial and final two seconds of the recordings were removed since they are subject to emission instabilities. The following acoustic parameters were analyzed: fundamental frequency (f0), Jitter percentage (%), pitch perturbation quotient (PPQ%), Shimmer percentage (%), amplitude perturbation quotient (APQ, %), noise harmonic ratio (NHR), and soft and phonation index (SPI).

### Moments studied

Preoperative (during the week of hospital admission), 1st (up to 15 days), 2nd (one month), 3rd (3 months), and 4th (6 months) postoperative evaluations, the latter being reserved only to patients who remained with alterations in the 3rd postoperative evaluation.

### Statistical analysis

#### Vocal acoustic analysis

The comparison between times was done using the Friedman test, complemented by the Dunn's multiple comparison test. To compare genders in each moment we used the Mann–Whitney test, considering a 5% significance level.

#### Videolaryngoscopy diagnoses, vocal symptoms, and correlation between vocal folds paralysis/paresis with surgery and histology results

These parameters were presented descriptively.

#### Correlation between histopathology and the type of surgical procedure

The chi square test was used to evaluate the homogeneity of the histopathology results and type of surgery. In addition, in order to identify differences between the proportions, the Goodman's test for contrasts between and within multinomial populations was used, considering 5% significance level. The test results are presented in the tables by upper and lower cases. Different capital letters in the same column indicate statistical difference (*p* < 0.05) between the proportions analyzed in the column. Different lower case letters in the same line indicate statistical difference (*p* < 0.05) between the proportions analyzed in the different populations.

## Results

### Age and gender

223 patients underwent thyroidectomy during the study period, but only 151 met the inclusion criteria and completed the evaluations, 130 women and 21 men; mean age 51.4 years (12–75 years).

### Vocal symptoms

**Figure 1:**
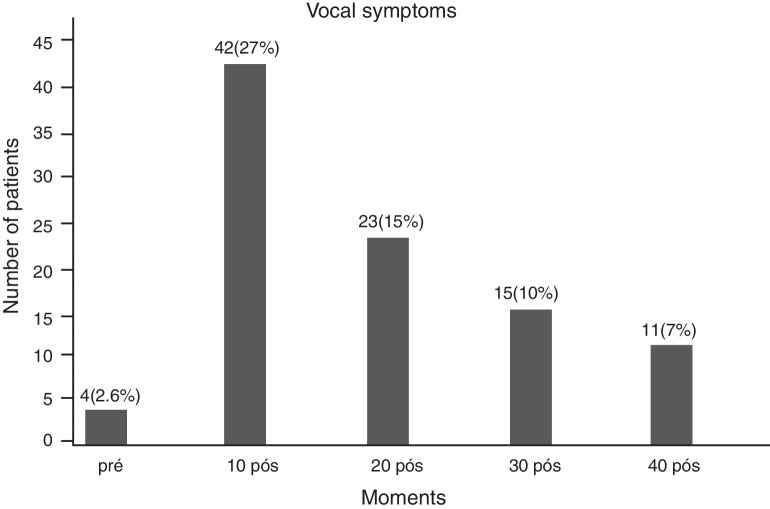
Vocal symptoms.

There was a predominance of the vocal symptoms in the first postoperative period (42 patients, 27%). There was a gradual decrease in symptoms at subsequent moments (Fig. 1).

### Acoustic vocal analysis

Table 1 depicts the results of acoustic voice analysis. The values of f0 decreased considerably in the three postoperative moments. These changes are most evident in women. APQ values also decreased in the three postoperative moments in women.

**Table 1:** Median (minimum and maximum) of acoustic parameters according to moments and gender.

Parameters	Gender	Moments				*p*-Value
		Pre	1st post	2nd post	3rd post	
F0	*F*	203.9^a^ (118.28–293.53)	193.3^b^ (76.47–293.53)	191.7^b^ (109.11–293.59)	196.0^b^ (86.19–276.05)	<0.00
	*M*	124.5 (85.10–156.61)	116.7 (0.00–158.21)	110.2 (91.02–186.92)	116.3 (89.47–186.92)	0.302
*p*-Value		<0.001	<0.001	<0.001	<0.001	

Jitter (%)	*F*	1.03 (0.27–6.54)	1.26 (0.32–18.31)	0.97 (0.25–10.54)	0.94 (0.18–5.22)	0.64
	*M*	1.09 (0.40–2.68)	1.09 (0.27–4.09)	0.71 (0.32–3.29)	1.28 (0.32–6.25)	0.133
*p*-Value		0.56	0.257	0.144	0.124	

PPQ	*F*	0.59 (0.15–3.78)	0.70 (0.19–13.59)	0.53 (0.14–6.57)	0.54 (0.11–2.93)	0.497
	*M*	0.63 (0.23–1.49)	0.63 (0.17–2.60)	0.43 (0.19–2.21)	0.74 (0.16–2.90)	0.133
*p*-value		0.507	0.341	0.218	0.111	

Shimmer (%)	*F*	3.24 (0.92–15.81)	3.34 (1.12–15.70)	3.28 (1.29–14.78)	3.10 (1.04–18.55)	0.211
	*M*	3.65 (1.74–16.43)	4.48 (1.83–11.49)	4.36 (1.42–12.16)	4.36 (2.68–16.32)	0.184
*p*-Value		0.529	0.081	0.157	0.006	

APQ	*F*	2.36^ab^ (0.70–9.57)	2.33^a^ (0.86–12.02)	2.28^ab^ (0.97–11.48)	2.12^b^ (0.76–12.50)	0.039
	*M*	3.04 (0.36–11.44)	3.30 (1.30–8.20)	3.30 (1.29–9.91)	3.07 (1.96–11.03)	0.183
*p*-Value		0.095	0.019	0.010	<0.001	

NHR	*F*	0.14 (0.05–0.32)	0.14 (0.07–0.50)	0.14 (0.07–0.40)	0.14 (0.07–0.48)	0.954
	*M*	0.15 (0.07–0.23)	0.14 (0.08–0.27)	0.14 (0.08–0.25)	0.14 (0.09–0.44)	0.995
*p*-Value		0.096	0.342	0.440	0.325	

SPI	*F*	9.31 (1.16–63.08)	9.17 (1.38–54.75)	8.86 (1.94–56.64)	9.34 (1.94–56.64)	0.313
	*M*	12.02 (5.89–31.71)	14.90 (5.36–42.08)	11.28 (3.06–40.67)	12.44 (0.59–30.62)	0.825
*p*-Value		0.041	0.021	0.103	0.183	

Different lower case letters indicate statistical difference (*p* < 0.05) between moments, separated by gender.

### Videolaryngoscopy

**Figure 2:**
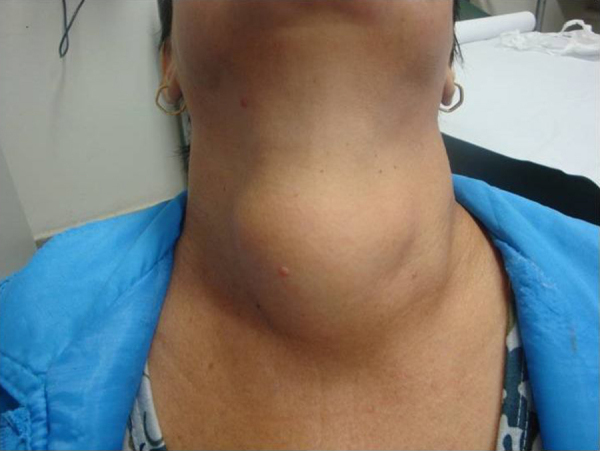
Large goiter in patients with hoarseness and respiratory distress.

**Figure 3:**
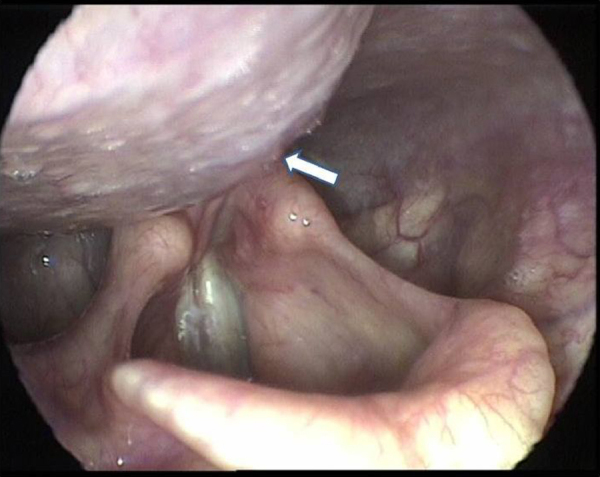
Hypopharyngeal projection of large goiter (ARROW).

The videolaryngoscopic findings are depicted in Table 2. Most of the patients had normal videolaryngoscopy in the preoperative period. In seven patients with very large goiter the endoscopic exams identified the projection of the goiter in the hypopharynx (Figure 2, Figure 3). These changes disappeared after surgery.

**Table 2:** Videolaryngoscopy findings in thyroidectomy patients in different moments.

Videolaryngoscopy	Moments				
	Pre *n* (%)	1st post *n* (%)	2nd post *n* (%)	3rd post *n* (%)	4th post *n* (%)
Normal	144 (95.3)	100 (66.2)	125 (82.8)	134 (88.7)	141 (93.4)
RLN paralysis	0 (0.0)	32 (21.2)	23 (15.2)	17 (11.3)	10 (6.6)
SLN paralysis	0 (0.0)	2 (1.3)	1 (0.7)	0 (0.0)	0 (0.0)
Projection to hypopharynx	7 (4.7)	0 (0.0)	0 (0.0)	0 (0.0)	0 (0.0)
Edema/hematoma	0 (0.0)	15 (10.0)	2 (1.3)	0 (0.0)	0 (0.0)
Granuloma	0 (0.0)	2 (1.3)	0 (0.0)	0 (0.0)	0 (0.0)
Total	151 (100.0)	151 (100.0)	151 (100.0)	151 (100.0)	151 (100.0)

Vocal folds paresis/paralysis (32 RLN injury and 2 SLN injury) were diagnosed in 34 patients in the 1st post. Of those, only ten (6.6%) remained with paralysis in the 4th postoperative evaluation. In only one case RLN paralysis was bilateral. RLN paralysis occurred on the left side in 12 patients, and on the right in 20 patients. In both SLN paresis/paralysis the affected side was the right and both recovered completely.

**Figure 4:**
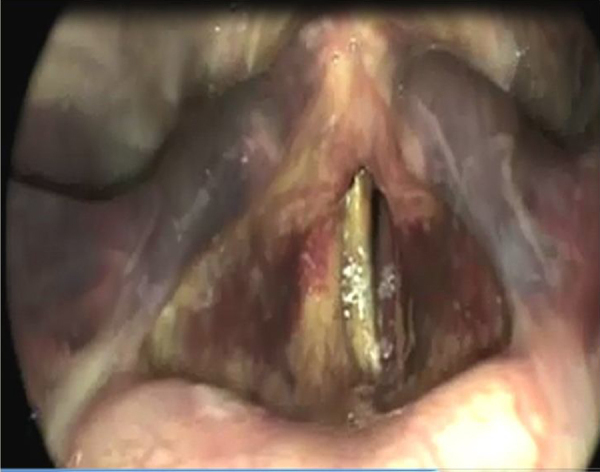
Laryngeal hematoma.

Hematomas (Fig. 4) and post-intubation granulomas were identified in 15 and 2 patients in the 1st postoperative evaluation, respectively. However none of these changes were observed after the 3rd post.

### Histopathology result and type of surgical procedure

The Table 3 shows the correlation between histopathology and type of surgery. Total thyroidectomy with and without lymph node dissection was performed in 111 patients, 65 with thyroid cancer (58.5%).

**Table 3:** Correlation between histopathology and type of surgery procedure.

Type of surgery *n* (%)	Histopathology *n* (%)			Total
	Colloid goiter	Thyroiditis	Cancer	
Lobectomy with isthmusectomy (LI)	33 (82.5)	5 (12.5)	2 (5.0)	40 (100.0)
Total thyroidectomy (TT)	43 (48.9)	3 (3.4)	42 (47.7)	88 (100.0)
Thyroidectomy + lymph node dissection (TT + LND)	0 (0.0)	0 (0.0)	23 (100.0)	23 (100.0)
Total	76 (50.3)	8 (5.3)	67 (44.4)	151(100.0)

Total thyroidectomy + lymph node dissection was not considered since it was pertinent only for cancer.

### Relationship between the type of surgery and vocal fold paralysis/paresis in the 1st postoperative evaluation

The relationship between the type of surgery and the occurrence of paralysis/paresis is depicted in Table 4. Among the 34 patients with vocal fold paralysis, 27 (79.4%) had undergone major surgical procedures.

**Table 4:** Correlation between the type of surgical procedure and vocal folds paralysis/paresis on the 1st postoperative evaluation.

Patients with paralysis/paresis	Types of surgery			
	LI *n* (%)	TT *n* (%)	TT + LND *n* (%)	Total *n* (%)
RLN	6 (17.6)	17 (50.0)	9 (26.6)	32 (94.2)
SLN	1 (2.9)	0 (0.0)	1 (2.9)	2 (5.8)
Total	7 (20.6)	17 (50.0)	10 (29.4)	34 (100.0)

LI, lobectomy + isthmusectomy; TT, total thyroidectomy; TT + LND, total thyroidectomy + lymph node dissection; RLN, recurrent laryngeal nerve; SLN, superior laryngeal nerve.

### Relationship between histopathology and vocal fold paralysis/paresis in the 1st postoperative evaluation

The relationship between the histopathology diagnosis and the occurrence of paralysis/paresis is depicted in Table 5. Among the 34 patients with vocal fold paralysis, 21 had thyroid cancer (61.8%) and 15 (38.2%) had benign goiters, seven of which were giant and extended into the hypopharynx.

**Table 5:** Correlation between histology and vocal cords paralysis/paresis in the 1st post.

Patients with paresis/paralysis	Histology			Total
	Goiter	Thyroiditis	Carcinoma	
RLN	12 (35.3)	0 (0.0)	20 (58.9)	32 (94.2)
SLN	1 (2.9)	0 (0.0)	1 (2.9)	2 (5.8)
Total	13 (38.2)	0 (0.0)	21 (61.8)	34 (100.0)

RLN, recurrent laryngeal nerve; SLN, superior laryngeal nerve.

## Discussion

In this study the symptoms were more prevalent in women in a ratio close to 6:1, as demonstrated by other authors,^1^, ^12^ and justified by the higher incidence of thyroid disease in women.

The delicate innervation of the thyroid gland maintains an intimate relationship with the structures of the larynx. Thus, vocal symptoms are frequent after thyroidectomy, and most of the time transient.^1^ In this study, vocal symptoms were reported by 42 patients (28%) in the 1st postoperative evaluation, decreasing considerably subsequently. These results can be attributed to laryngeal disorders diagnosed by videolaryngoscopy in the immediate postoperative period, such as paralysis, hematomas, and granulomas, most with remission in subsequent postoperative evaluations.

Some authors had reported higher incidence of voice disorders in the postoperative period of thyroidectomy than those presented in the current series. Soylu et al.^5^ evaluated the vocal quality of 48 thyroidectomy patients (*n* = 8 lobectomy; *n* = 40 total thyroidectomy) in three moments (preoperatively, 2nd postoperatively and after 3 months). The authors reported vocal changes in 37.5% of patients in the early postoperative period that persisted after 3 months in 14.6%. F0 was the only acoustic parameter that remained altered after 3 months. In the early postoperative period, changes in f0 were more significant in patients submitted to total thyroidectomy.

Page et al.^13^ conducted a subjective voice analysis in 395 thyroidectomy patients (*n* = 340 multinodular goiter; *n* = 25 Graves’ disease; *n* = 20 thyroid cancer). The voices were classified as: hoarse, low or weak pitch and voice fatigue. Patients who had inferior laryngeal nerve paralysis were excluded. The authors identified 87 patients (21%) with abnormal voice at the preoperative evaluations and 151 patients (49%) had voice impairment after surgery. Out of the 87 patients who had abnormal voices at the preoperative evaluation, only eight patients kept an impaired voice after one year. Among the possible causes, the authors highlight the modifications in the resonator channel caused by the cervical bulky goiter extending to the retropharyngeal space as registered by us during videolaryngoscopic exams. Of the 151 patients with abnormal postoperative voice, 46% recovered within one month and after one year, only 5 patients (3%) still had abnormal voice.

Park et al.^14^ analyzed the voice of 217 patients who underwent thyroidectomy in the pre and postoperative moments (2 weeks, and 3, 6, and 12 months). Significant decreased pitch were evident in 93 (42.85%) patients after surgery, especially in the first 6 months after surgery, and only 18.4% of patients had lower-pitched voices one year after surgery. The voice changes of patients submitted to total thyroidectomy were significantly higher than those who underwent lobectomy at 2 week after surgery, but did not differ at the 3, 6, and 12 month follow-ups.

Among the various causes of post thyroidectomy dysphonia the most important are endotracheal intubation, manipulation, surgical stretching and fixation of the cervical muscles, laryngeal nerves and cricothyroid muscle injuries. Functional dysphonia can occur even without injuries to the laryngeal nerves. Maeda et al.^8^ evaluated the voices of 110 patients after TT with no nerve damage and found decreased Maximum Phonation Time and f0, and increase in the other acoustic parameters, especially in patients with greater surgical manipulation. Pedro Netto et al.^4^ evaluated 100 patients after partial (*n* = 42) or total thyroidectomy (*n* = 58) and found vocal changes in 29.7% with no paralysis, representing functional dysphonia. Paralysis was diagnosed in 10 patients, of which only 5% complained of dysphonia.

In an interesting systematic review conducted by Lang et al.^15^ included 896 patients after TT and identified decreased f0 and increased shimmer and NHR in the immediate postoperative period, especially in men, confirming the results of many studies.

Among the abnormalities detected in the preoperative videolaryngoscopies of this study, we emphasize the projection of bulkier goiters into the hypopharyngeal region, compressing local structures (Figure 1, Figure 2), seen in seven patients. Page et al.^13^ point out that the hypopharyngeal bulging caused by goiter modifies the resonator channel and changes vocal quality, justifying part of the alterations recorded in subjective voice assessment.

Vocal fold paralysis was diagnosed in 34 patients in the 1st postoperative evaluation, 32 (21%) with RLN injury and 2 (1.3%) with SLN injury. In the 6 month follow up exam, only 10 of them presented paralysis/paresis. These values indicate a high rate of nerve function recovery over the months, reducing the chances of permanent paralysis. In addition to the previously mentioned causes for vocal fold paralysis post thyroidectomy, we must highlight the fact that these surgeries were performed in a university hospital, a place of teaching and training of residents, collaborating with the highest percentages. According to some authors, RLN injuries range from 1% to 13%.^5^, ^6^, ^8^, ^16^ For SLN, the range of values is more extensive, between 2% and 30%.^3^, ^4^, ^6^, ^10^, ^17^ We believe that these results can be attributed to difficulties in the diagnosis of SLN paralysis, demanding attention and experience of the examiner during the exam, since the mobility of the vocal fold is preserved and only its tension is decreased.

The reported risk factors of laryngeal nerve damage in thyroidectomy are: goiter grater than 5 cm, patients older than 50 years, reoperation, malignant disease, type of surgery (partial or total thyroidectomy, with or without lymph node dissection), and surgeon experience.^7^, ^16^, ^17^ Other causes include direct damage (mechanical or thermal) during surgery, perineural vascular injury, and compression by hematoma. Many studies interrupt follow up at three months; however, our results and other authors’ have shown that vocal symptoms and laryngeal paralysis greatly reduce after six months. It is, therefore, advisable to extend the follow-up time before proposing new surgeries.^18^, ^19^, ^20^, ^21^ For Christou and Mathonnet^7^ temporary dysphonia secondary to RLN injury occur in 5–18% of cases, and permanent dysphonia in only 1–3.5%. These authors point out that less than 0.5% paralyzes are bilateral, also found in our study.

In this study, benign thyroid diseases accounted for approximately 50% of all cases and cancer for 44%, the latter requiring more extended surgical procedures (TT and TT + LND). These surgeries accounted for 79% of vocal folds paralysis/paresis. These rates are similar to statistics presented by other authors.^3^, ^4^, ^6^, ^14^, ^16^, ^18^

Injuries to the RLN cause symptoms of vocal asthenia and low voice; however, SLN injuries may go unnoticed because the voice symptoms are more frequent in women and voice professionals. Chun et al.^6^ evaluated 300 thyroidectomy patients by videolaryngoscopy, vocal acoustic analysis and auditory-perceptual analysis. They identified 31 patients (10.3%) with postoperative RLN paralysis and 54 (18%) with difficulties in sustaining the pitch. The voice quality questionnaires were more accurate in identifying abnormal voices, corresponding to 91.6% of patients in the postoperative period, this vocal assessment tool being highly valued by the authors.

We emphasize the importance of adopting simple measures such as standardization of routine videolaryngoscopy and vocal analysis before and after surgery, since they enable early diagnosis of voice disorders and monitoring the progress of laryngeal lesions, particularly paralyzes.

## Conclusions

Vocal symptoms were reported by 27.8% of our patients in the 1st postoperative evaluation after thyroidectomy, reducing to 7% in 6 months. In the acoustic analysis f0 and APQ were decreased. Temporary paralysis of the vocal folds secondary to recurrent laryngeal nerve damage occurred in 21% of the patients, persisting after 6 months in only 6.6% of cases. Temporary paralysis consequent to superior laryngeal nerve damage occurred in 1.3%, with complete recovery in all cases within 6 months.

## Conflicts of interest

The authors declare no conflicts of interest.
